# Statin Use and In-hospital Mortality in Patients with COVID-19 and Coronary Heart Disease

**DOI:** 10.1038/s41598-021-02534-2

**Published:** 2021-12-13

**Authors:** Lan Shen, Lin Qiu, Li Wang, Hengye Huang, Dong Liu, Ying Xiao, Yi Liu, Jingjin Jin, Xiulan Liu, Dao Wen Wang, Ben He, Ning Zhou

**Affiliations:** 1 Department of Cardiology, Clinical Research Unit, Shanghai Chest Hospital, Shanghai Jiaotong University, Shanghai, 200030 China; 2 Department of Pharmacy, Tongji Hospital, Tongji Medical College, Huazhong University of Science and Technology, 1095 Jiefang Ave., Wuhan, 430030 China; 3 Department of Geriatrics, School of Medicine, Shanghai Renji Hospital, Shanghai Jiaotong University, Shanghai, 200127 China; 4 School of Public Health, School of Medicine, Shanghai Jiaotong University, Shanghai, 200025 China; 5 Division of Cardiology, Department of Internal Medicine, Tongji Hospital, Tongji Medical College, Huazhong University of Science and Technology, 1095 Jiefang Ave, Wuhan, 430030 China

**Keywords:** Cardiology, Diseases, Medical research

## Abstract

The worsening progress of coronavirus disease 2019 (COVID-19) is attributed to the proinflammatory state, leading to increased mortality. Statin works with its anti-inflammatory effects and may attenuate the worsening of COVID-19. COVID-19 patients were retrospectively enrolled from two academic hospitals in Wuhan, China, from 01/26/2020 to 03/26/2020. Adjusted in-hospital mortality was compared between the statin and the non-statin group by CHD status using multivariable Cox regression model after propensity score matching. Our study included 3133 COVID-19 patients (median age: 62y, female: 49.8%), and 404 (12.9%) received statin. Compared with the non-statin group, the statin group was older, more likely to have comorbidities but with a lower level of inflammatory markers. The Statin group also had a lower adjusted mortality risk (6.44% vs. 10.88%; adjusted hazard ratio [HR] 0.47; 95% CI, 0.29–0.77). Subgroup analysis of CHD patients showed a similar result. Propensity score matching showed an overall 87% (HR, 0.13; 95% CI, 0.05–0.36) lower risk of in-hospital mortality for statin users than nonusers. Such survival benefit of statin was obvious both among CHD and non-CHD patients (HR = 0.30 [0.09–0.98]; HR = 0.23 [0.1–0.49], respectively). Statin use was associated with reduced in-hospital mortality in COVID-19. The benefit of statin was both prominent among CHD and non-CHD patients. These findings may further reemphasize the continuation of statins in patients with CHD during the COVID-19 era.

## Introduction

Syndrome of acute respiratory stress coronavirus 2 (SARS-COV-2) has infected more than 200 million cases, with more than 4.26 million deaths has been reported^1^. The severity of COVID-19 is attributed to the inflammatory status, presented as an increased inflammatory biomarker such as C-Reactive Protein (CRP), Interleukin 2 (IL-2), and Interleukin 6 (IL-6), which are predictors of mortality^2^. Statin, a well-known anti-inflammatory medication, has been suggested as the possible medication to attenuate the worsening inflammatory reactions during COVID-19^3^. However, animal studies showed that statin increases membrane-bound angiotensin-converting enzyme two receptors, the port of entry for COVID-19 virus. Clinicians are also concerned about the assumption that statin may enhance the virus entry into the human cells^4,5^. Consequently, there are fears that patients may discontinue statin during COVID-19 treatment.

Pre-existing coronary heart disease (CHD) predisposes patients to susceptibility to COVID-19 and serves as one of the strongest predictors in COVID-19 mortality^6^. Cardiomyocyte are specific target for SARS-CoV-2^7^. SARS-CoV-2 infected the cardiomyocyte directly by virus interaction with angiotensin-converting enzyme 2 (ACE2) receptor which was activated by transmembrane protease serine protease-2 (TMPRSS2)^8^, enhanced virus's entrance and replication^9–11^. Moreover, common comorbidity of CHD such as diabetes increases over-inflammation, inflammatory/oxidative stress, cytokine storm following virus entrance and replication, and the extent of cytokine storm was associated with disease severity^12–14^. Previous studies showed that statin protected diabetic COVID-19 patients from in-hospital mortality by anti-inflammatory properties and antiplatelet effect^3^. CHD is highly prevalent in COVID-19 patients, especially severe patients^15^. Statin was the most widely used medication among CHD patients and is well known for its anti-inflammatory effect and effect in reducing reactive oxygen species and platelet reactivity. Our study sought to determine if statin use is associated with in-hospital survival benefits among COVID-19, especially in CHD patients.

## Material and methods

### Study design and participants

This retrospective, multi-center study included 3133 patients with COVID-19. All patients were admitted to two academic hospitals, the Sino-French new city branch and the Optical Valley branch affiliated with Tongji Hospital, Tongji Medical College, Huazhong University of Science and Technology. Both centers were exclusively managed COVID-19 patients in Wuhan, China. Tongji Hospital Ethics Commission approved the study. Written informed consent was waived because of the retrospective nature of the study by the Ethical Committee of Tongji Hospital, Tongji Medical College, Huazhong University of Science and Technology.

Inclusion criteria were patients who were 18 years of age or older, with COVID-19 admitted from January 26 to April 30, 2020. According to the New Coronavirus Pneumonia Prevention and Control Program (7th edition) published by the National Health Commission of China and WHO guidance, the diagnosis of COVID-19 was confirmed by qualitative Reverse Transcription Polymerase Chain Reaction (RT-RNA) tests on a nose/ throat swab positive for COVID-19^1,16^. Patients with incomplete medical records (e.g., transfer to other hospitals) were excluded from the analysis. Patients with contraindications for statins use, including presented serum levels of CK or aminotransferase of more than five times the upper limit of normal (ULN) at admission, were also excluded.

### Data collection

Electronic medical records of hospitalized patients with COVID-19 were reviewed to extract information on demographic information, history of comorbidities, clinical characteristics, laboratory data on admission, seven days post-admission and their peak value, therapeutic interventions (antiplatelet therapy, beta-blockers, angiotensin-converting enzyme inhibitors [ACEI] and ARBs [ARB], Calcium Channel Blocker [CCB], antiviral treatment, immunological treatment, ventilation, and Extracorporeal membrane oxygenation [ECMO]), vasopressor medication such as dopamine, epinephrine, and norepinephrine during the hospitalization, and in-hospital mortality. The patient demographic information (age and sex), laboratory data on admission (blood cell count, D-Dimer, organ function markers, serum electrolyte, and inflammatory marker Interleukin-6 [IL-6], C-Reactive Protein [CRP], Procalcitonin [PCT]), laboratory data seven days post-admission and their peak value were collected from the laboratory information system. Comorbidities were extracted from medical history. The symptom's time to hospital presentation, length of stay, and in-hospital treatments were collected from medical records. All data were double-checked against source documents by two independent researchers.

### Definition

The severity of COVID-19 was classified according to the diagnosis and treatment scheme for COVID-19 of China (7th edition)^16^. CHD patients were defined as those who had a medical record shown CHD as one of the comorbidities in the discharge diagnosis by clinicians. Patients were classified as statin group if medical history showed statin administration within the first three days post-hospital admission.

### Statistical analysis

Continuous variables were expressed as the median and interquartile range (IQR), and categorical variables were expressed as number and percentage (%). Statistical differences between the two groups were analyzed by the Mann–Whitney U test for continuous variables, while categorical variables were compared by Fisher's exact test or χ2 test. The risk of in-hospital mortality and the corresponding hazards ratio (HR) were calculated using the multivariable Cox regression model to compare the statin group versus the non-statin group. A full model included demographics, clinical and laboratory variables independently associated with statin use or in-hospital mortality at a P < 0.05. The final multivariable adjustment including statin use (yes or no), age, sex, history of comorbidities, respiratory rate, severity classification, Lab results including serum B-type natriuretic peptide (BNP), Troponin I (TNI), aspartate aminotransferase (AST), Alanine transaminase (ALT), Creatinine (Cr), K, Na, Ca, glucose, Albumin, Lactate dehydrogenase (LDH), White blood cell (WBC), Lymphocyte, Neutrocyte, Platelet, CRP (C Reactive Protein), Procalcitonin (PCT), Interleukin 6 (IL-6), Interleukin 2 (IL-2), Tumor necrosis factor-α (TNF-α), activated partial thromboplastin time (APTT), international normalized ratio (INR), D-Dimer and Total Cholesterol (TC), vasoactive drugs, ARB and beta-blocker (BB) use during hospitalization. A two-side α less than 0.05 was considered statistically different. Data were analyzed in R-3.6.3 (R Foundation for Statistical Computing, Vienna, Austria) and SPSS Statistics (version 22.0, IBM, Armonk, NY, USA).

### Propensity score-matched analysis

To minimize baseline differences between statin and non-statin groups, we performed propensity score-matched analysis (PSM). Baseline matching variables included age, sex, history of comorbidities, Na, Albumin, Glucose, Hemoglobin, Lymphocyte, Platelet, CRP, IL-6, TC, serum troponin I (TNI) levels, severity classification, use of vasoactive medicine, antiplatelet therapy, ACEI, ARB, beta-blocker, and CCB. To further balance the baseline difference between statin and non-statin groups in subgroups of the CHD and non-CHD populations, we performed PSM again in the CHD and non-CHD groups, respectively. Residual imbalance (p < 0.05) between statin and the non-statin group was further adjusted in the Cox regression model.

Statin users and nonusers were paired at 1:1 according to the propensity scores using exact matching with a caliper size of 0.05. The balance of covariates was evaluated by p-value before and after matching, and p-value >  = 0.05 was considered qualified balancing between the two groups. For subgroup study of CHD or non-CHD population, the pair ratio was also 1:1 between statin and non-statin group, with the caliper size as 0.05.

## Result

1. Patient Characteristics
  Overall, 3133 patients (62 ± 19 years old; 49.8% female) were admitted with COVID-19. CHD (7.9%), Diabetes mellitus (13.2%), hypertension (317%), and Chronic obstructive pulmonary (6.4%) were highly prevalent comorbidities. On average, patients presented 13 (IQR, 12) days after the onset of symptoms and had elevated inflammatory markers on admission including CRP (11.5; IQR, 56.9 mg/dL), IL-6 (5.0; IQR, 19.5 ng/mL) and PCT (0.06; IQR, 0.07 ng/mL) (Table 1).
2. Statin Use and In-Hospital Mortality
  Among all admitted patients, 12.9% (n = 404) received a statin. Those on statin therapy were older (67 ± 15 vs. 61 ± 20 years; p < 0.001) and had more likely to have comorbidities such as CHD, diabetes, cerebrovascular disease, dyslipidemia, hypertension, heart failure, and atrial fibrillation. (All p < 0.001) Despite a higher comorbidity burden, we observed lower inflammatory markers such as at presentation a lower IL-2 (523.5, IQR [422.25] vs. 583.5, IQR [579.25], p = 0.001), seven days later a lower IL-2 (431, IQR [328.5] vs. 504.5, IQR [443.5], p < 0.001) and lower IL-2 peak level (551.5, IQR [431.75] vs. 606, IQR [630.75], p < 0.001). Also, patients on a statin had a similar D-Dimer to start with but a lower D-Dimer 7 days later (0.49, IQR [0.8] vs. 0.63, IQR [1.35], p = 0.006). (Table 1).
  Meanwhile, we observed lower cumulative in-hospital mortality in those who were receiving a statin (6.44% versus 10.88%; P < 0.01, unadjusted HR 0.46 [0.31, 0.69]). Further analysis revealed that this effect was prominent both in CHD and non-CHD patients. Patients on a statin had a lower cumulative in-hospital mortality both with CHD (10.4% versus 26%; p = 0.001;) or without CHD (4.7% versus 10.2%; p = 0.003). (Table 2).
  In the logistic regression model, after adjusting for age, sex, comorbidities, lab results on admission, including inflammatory markers, in-hospital medications, and severity classification, the use of statin was associated with lower all-cause mortality (adjusted HR, 0.47; 95% CI, 0.29–0.77) versus non-statin group. The results remained consistent and statistically significant with a propensity score matching analysis, demonstrating a lower risk of in-hospital mortality in the statin group (adjusted HR, 0.13; 95% CI [0.05–0.36]). (Table 2).
3. CHD and Statin Use
  Among patients with CHD, 125 (50.4%) received, and 123 (49.6%) did not receive a statin. Those on a statin were at a similar age, with the similar possibility to have comorbidities such as hypertension, dyslipidemia, heart failure, diabetes, and pulmonary disease, as well as a similar COVID-19 severity classification. However, patients on statin were more likely to have concomitant use of aspirin, clopidogrel, beta-blocker, ARB, and CCB (all p < 0.05). As for in-hospital treatment, those on statin were more likely to have concomitant use of aspirin, clopidogrel, beta-blocker, ARB, and CCB, but less likely to use vasoactive drugs (12[9.6%] vs. 23[18.7%], all p < 0.05). For inflammatory markers, those receiving a statin had a similar CRP but lower IL-6 (8.35, IQR [21.15] vs. 10.89, IQR [28.09], p = 0.036) at presentation. Seven days later, patients on statin had both lower CRP (2.7, IQR [13.6] vs. 4.6, IQR [53.9], p = 0.005) and lower IL-6 (4.96, IQR [11.08] vs. 9.5, IQR [40.74], p = 0.023). When examining the peak level of CRP and IL-6, patients on a statin had a similar CRP level and a lower IL-6 level (10.52, IQR [29.49] vs. 11.69, IQR [47.46], p = 0.02), compared with those off statin. Also, patients on statin had a similar D-Dimer at presentation but a lower D-Dimer 7 days later (0.49, IQR [0.8] vs. 0.63, IQR [1.35], 0.006). (Table 3)
4. Statin Use and In-Hospital Mortality in CHD patients.
  There was a 54% reduction in in-hospital mortality (unadjusted HR 0.28, 95% CI 0.15–0.54; adjusted HR 0.46, 95% CI 0.23–0.90) associated with patients on statin with CHD admitted with COVID-19 in the multivariable risk regression model. Through PSM, statin recipients and nonrecipients had a well-balanced baseline characteristic, including age, sex, comorbidities, lab results, concomitant medications, and severity classification. (Table 4) The result remained statistically significant after PSM, and statin was associated with reduced in-hospital mortality both in CHD (adjusted HR 0.30, 95%CI 0.09–0.98) and non-CHD patients (adjusted HR 0.23, 95%CI 0.1–0.49). (Table 3).

**Table 1:** Baseline characteristics of the study population by statin use before and after PSM.

	Before PSM (N = 3133)				After PSM (N = 604)			
	All	Statin (N = 404)	Non-Statin (N = 2729)	p	All	Statin (N = 302)	Non-Statin (N = 302)	p
Age (years)	62 (19)	67 (15)	61 (20)	< 0.001	66 (16)	66 (15.25)	65 (17)	0.264
**Sex (%)**								
Male	1572 (50.18%)	212 (52.48%)	1360 (49.84%)	0.322	312 (51.66%)	164 (54.3%)	148 (49.01%)	0.193
Female	1561 (49.82%)	192 (47.52%)	1369 (50.16%)		292 (48.34%)	138 (45.7%)	154 (50.99%)	
**Comorbidities (%)**								
Hypertension	1159 (36.99%)	234 (57.92%)	925 (33.9%)	< 0.001	293 (48.51%)	158 (52.32%)	135 (44.7%)	0.061
Dyslipidemia	23 (0.73%)	12 (2.97%)	11 (0.4%)	< 0.001	13 (2.15%)	5 (1.66%)	8 (2.65%)	0.401
CHD	248 (7.92%)	125 (30.94%)	123 (4.51%)	< 0.001	102 (16.89%)	62 (20.53%)	40 (13.25%)	0.017
Aortic Dissection	4 (0.13%)	2 (0.5%)	2 (0.07%)	0.027	4 (0.66%)	2 (0.66%)	2 (0.66%)	1
Heart Failure	33 (1.05%)	13 (3.22%)	20 (0.73%)	< 0.001	12 (1.99%)	6 (1.99%)	6 (1.99%)	1
Atrial fibrillation	20 (0.64%)	9 (2.23%)	11 (0.4%)	< 0.001	11 (1.82%)	5 (1.66%)	6 (1.99%)	0.761
Other arrhythmia	333 (10.63%)	46 (11.39%)	287 (10.52%)	0.597	56 (9.27%)	37 (12.25%)	19 (6.29%)	0.012
Diabetes milieus	414 (13.21%)	105 (25.99%)	309 (11.32%)	< 0.001	131 (21.69%)	65 (21.52%)	66 (21.85%)	0.922
Lung disease	199 (6.35%)	27 (6.68%)	172 (6.3%)	0.77	41 (6.79%)	17 (5.63%)	24 (7.95%)	0.258
Renal insufficiency	69 (2.2%)	12 (2.97%)	57 (2.09%)	0.26	20 (3.31%)	10 (3.31%)	10 (3.31%)	1
Stroke	102 (3.26%)	38 (9.41%)	64 (2.35%)	< 0.001	41 (6.79%)	20 (6.62%)	21 (6.95%)	0.872
Tumor	129 (4.12%)	13 (3.22%)	116 (4.25%)	0.33	23 (3.81%)	10 (3.31%)	13 (4.3%)	0.524
Liver disease	55 (1.76%)	2 (0.5%)	53 (1.94%)	0.039	5 (0.83%)	2 (0.66%)	3 (0.99%)	0.654
**Lab on admission**								
BNP (pg/ml)	85 (327)	118.5 (640.75)	82 (289)	0.248	99 (459.25)	93 (491)	130 (433)	0.819
Hs-cTnI (ng/ml)	4 (6.6)	6.15 (7.18)	3.7 (6.2)	0.459	5.3 (7.2)	5 (7.1)	5.55 (7.43)	0.051
ALT (u/l)	23 (24)	24 (21)	22 (24)	0.546	24 (23.75)	24 (22)	23 (27)	0.71
AST (u/l)	25 (19)	25 (16)	25 (19)	0.312	25 (18)	24.5 (16)	25 (20)	0.244
Creatinine (μmol/l)	68 (27)	70 (26)	68 (28)	0.714	69 (26)	69 (26)	69 (26)	0.298
K (mmol/L)	4.19 (0.66)	4.19 (0.77)	4.19 (0.64)	0.296	4.17 (0.72)	4.23 (0.8)	4.16 (0.67)	0.571
Na (mmol/L)	139.9 (4.2)	139.55 (5.07)	139.9 (4.1)	< 0.001	139.6 (4.6)	139.6 (5.1)	139.55 (4.03)	0.525
Ca (mmol/L)	2.16 (0.17)	2.15 (0.16)	2.16 (0.17)	0.347	2.16 (0.17)	2.16 (0.16)	2.15 (0.17)	0.427
Glucose (mmol/L)	5.91 (2.37)	6.17 (2.75)	5.87 (2.27)	0.001	6.09 (2.46)	6.06 (2.53)	6.11 (2.23)	0.836
ALB (g/L)	36.3 (8.3)	35.6 (7.58)	36.4 (8.4)	0.07	35.5 (7.68)	35.9 (7.45)	35.1 (8)	0.301
LDH (U/L)	248 (143)	258 (129)	247 (145)	0.316	255 (132.75)	247 (114.25)	260.5 (161.25)	0.165
Hb (g/l)	127 (22)	124 (21)	127 (22)	0.05	125 (22)	124 (20.25)	126 (24)	0.558
WBC (× 10^9^/l)	5.84 (3.01)	6.08 (2.91)	5.81 (2.98)	0.195	6.03 (2.9)	6.06 (2.88)	6.01 (2.73)	0.458
Lymph (× 10^9^/l)	1.2 (0.87)	1.19 (0.8)	1.2 (0.88)	0.381	1.2 (0.84)	1.25 (0.82)	1.12 (0.85)	0.074
PLT (× 10^9^/l)	221 (117)	226 (135.5)	220 (115)	0.013	226 (129.75)	229.5 (131.5)	225 (126.25)	0.477
hs_CRP (mg/l)	11.5 (56.9)	12.9 (51.53)	11.3 (57.3)	0.353	14.15 (57.15)	11.15 (53)	19.2 (62.83)	0.406
IL-2 (pg/ml)	526 (488.5)	526 (424.5)	525 (495.5)	0.196	552.5 (492)	523.5 (422.25)	583.5 (579.25)	0.001
IL_6 (pg/ml)	4.98 (19.46)	6.35 (15.88)	4.84 (19.73)	0.278	6.45 (19)	5.91 (15.01)	7.17 (23.85)	0.478
TNF-α (ng/ml)	8 (4.5)	8.3 (4.3)	7.9 (4.5)	0.586	8.3 (4.35)	8.1 (4.25)	8.6 (4.43)	0.101
PCT (ng/ml)	0.06 (0.07)	0.06 (0.06)	0.06 (0.07)	0.245	0.07 (0.07)	0.06 (0.06)	0.07 (0.08)	0.91
APTT (s)	38.6 (6.5)	38.5 (6.38)	38.7 (6.5)	0.103	38.6 (6.65)	38.55 (6.35)	38.7 (7)	0.761
PT_INR (s)	1.06 (0.12)	1.05 (0.13)	1.06 (0.12)	0.379	1.06 (0.13)	1.05 (0.13)	1.06 (0.12)	0.283
D_Dimer (μg/ml)	0.67 (1.42)	0.78 (1.41)	0.66 (1.4)	0.85	0.78 (1.58)	0.69 (1.4)	0.87 (1.71)	0.509
TC (mmol/L)	3.82 (1.25)	3.71 (1.5)	3.83 (1.23)	0.224	3.77 (1.36)	3.78 (1.51)	3.77 (1.23)	0.26
**7-days after admission**								
APTT (s)	38.2 (6.2)	38.5 (6.18)	38.2 (6.2)	0.492	38.4 (6.5)	38.4 (6.23)	38.4 (6.93)	0.096
PT_INR (s)	1.04 (0.11)	1.04 (0.12)	1.04 (0.11)	0.525	1.04 (0.11)	1.04 (0.11)	1.04 (0.12)	0.172
D_Dimer (μg/ml)	0.52 (1.02)	0.56 (0.96)	0.51 (1.04)	0.047	0.56 (1.02)	0.49 (0.8)	0.63 (1.35)	0.006
hs_CRP (mg/l)	2.2 (8)	2.2 (8.1)	2.2 (8)	0.032	2.2 (6.75)	1.95 (5.75)	2.4 (9.15)	0.021
IL-2 (pg/ml)	450 (384)	443.5 (334.5)	451 (390)	0.088	460 (371.5)	431 (328.5)	504.5 (443.5)	< 0.001
IL_6 (pg/ml)	3.27 (7.97)	3.72 (8.37)	3.2 (7.95)	0.077	3.6 (7.9)	3.2 (6.46)	4.13 (9.71)	0.2
TNF-α (ng/ml)	7.7 (4.6)	8.2 (4.6)	7.7 (4.7)	0.591	8.1 (4.5)	8.1 (4.43)	8.1 (4.63)	0.566
TC (mmol/L)	4.23 (1.41)	3.8 (1.54)	4.27 (1.36)	< 0.001	4.03 (1.5)	3.98 (1.54)	4.07 (1.44)	0.391
**Lab peak**								
IL6 peak (pg/ml)	6.27 (24.83)	7.59 (22.93)	6.03 (25.19)	0.066	7.45 (22.55)	6.8 (17.87)	8.85 (29.44)	0.13
IL2 peak (pg/ml)	553 (509)	554 (438.25)	553 (520.5)	0.099	569.5 (512.25)	551.5 (431.75)	606 (630.75)	< 0.001
TNF-αpeak (ng/ml)	8.7 (5)	8.9 (4.65)	8.6 (4.9)	0.495	8.95 (4.78)	8.8 (4.53)	9 (4.83)	0.327
CRP peak (mmol/L)	14.2 (61.55)	16.6 (58.68)	13.9 (62.5)	0.397	17.55 (61.8)	14.25 (58.38)	22 (67.78)	0.359
**Meds (%)**								
Norepinephrine	237 (7.56%)	28 (6.93%)	209 (7.66%)	0.606	38 (6.29%)	12 (3.97%)	26 (8.61%)	0.019
Epinephrine	342 (10.92%)	37 (9.16%)	305 (11.18%)	0.225	61 (10.1%)	18 (5.96%)	43 (14.24%)	0.001
Dopamine	200 (6.38%)	25 (6.19%)	175 (6.41%)	0.863	34 (5.63%)	12 (3.97%)	22 (7.28%)	0.078
Dobutamine	36 (1.15%)	5 (1.24%)	31 (1.14%)	0.858	10 (1.66%)	4 (1.32%)	6 (1.99%)	0.524
Aspirin	269 (8.59%)	172 (42.57%)	97 (3.55%)	< 0.001	183 (30.3%)	89 (29.47%)	94 (31.13%)	0.659
Clopidogrel	142 (4.53%)	101 (25%)	41 (1.5%)	< 0.001	87 (14.4%)	47 (15.56%)	40 (13.25%)	0.418
Ticagrelor	14 (0.45%)	12 (2.97%)	2 (0.07%)	< 0.001	6 (0.99%)	4 (1.32%)	2 (0.66%)	0.413
Methylprednisolone	873 (27.86%)	103 (25.5%)	770 (28.22%)	0.255	158 (26.16%)	77 (25.5%)	81 (26.82%)	0.712
ACEI	92 (2.94%)	35 (8.66%)	57 (2.09%)	< 0.001	33 (5.46%)	19 (6.29%)	14 (4.64%)	0.372
ARB	348 (11.11%)	110 (27.23%)	238 (8.72%)	< 0.001	117 (19.37%)	66 (21.85%)	51 (16.89%)	0.123
Βeta-blocker	91 (2.9%)	35 (8.66%)	56 (2.05%)	< 0.001	23 (3.81%)	15 (4.97%)	8 (2.65%)	0.137
CCB	415 (13.25%)	92 (22.77%)	323 (11.84%)	< 0.001	112 (18.54%)	61 (20.2%)	51 (16.89%)	0.296
Non-invasive ventilator (%)	375 (11.97%)	57 (14.11%)	318 (11.65%)	0.156	81 (13.41%)	40 (13.25%)	41 (13.58%)	0.905
Invasive Ventilator (%)	285 (9.1%)	38 (9.41%)	247 (9.05%)	0.817	50 (8.28%)	27 (8.94%)	23 (7.62%)	0.556
ECMO (%)	27 (0.86%)	4 (0.99%)	23 (0.84%)	0.765	5 (0.83%)	3 (0.99%)	2 (0.66%)	0.654
**Type (%)**								
1	888 (28.34%)	69 (17.08%)	819 (30.01%)	< 0.001	145 (24.01%)	58 (19.21%)	87 (28.81%)	0.272
2	1175 (37.5%)	173 (42.82%)	1002 (36.72%)		227 (37.58%)	132 (43.71%)	95 (31.46%)	
3	1070 (34.15%)	162 (40.1%)	908 (33.27%)		232 (38.41%)	112 (37.09%)	120 (39.74%)	

PSM, propensity score match; CHD, coronary vascular disease; BNP, serum B-type natriuretic peptide; TNI, Troponin I; ALT, Alanine Aminotransferase; AST, Aspartate transaminase; Cr, Creatinine; ALB, Albumin; LDH, Lactate dehydrogenase; WBC, White blood cell; Lymph, Lymphocyte; Plt, Platelet; CRP, C Reactive Protein; IL-2, Interleukin 2; IL-6, Interleukin 6; TNF-α, P.C.T., Procalcitonin; Tumor necrosis factor-α; APTT, activated partial thromboplastin time; INR, international normalized ratio; TC, Total Cholesterol; ACEI, Angiotensin-converting-enzyme inhibitors; ARB, angiotensin receptor blockers; CCB, Calcium Channel Blocker; ECMO, Extracorporeal membrane oxygenation.

Continuous variables are presented as median (IQR), categorical variables are presented as n percentage.

**Table 2:** In-Hospital mortality in CHD and Non-CHD patients before and after the PSM.

Statin vs. non-statin	Crude mortality rate			Unmatched	Unmatched	Matched
	Statin	Non-statin	p	Unadjusted HR.* (95% CI) Statin vs. Nonstatin	Adjusted HR. (95% CI) Statin vs. Nonstatin	Adjusted HR. (95% CI) Statin vs. Nonstatin
Overall	6.44%	10.88%	< 0.001	0.46 (0.31,0.69)	0.47 (0.29,0.77)	0.13 (0.05,0.36)
CHD	13 (10.4%)	32 (26.0%)	0.001	0.28 (0.15,0.54)	0.46 (0.23,0.90)	0.30 (0.09,0.98)
Non-CHD	13 (4.7%)	265 (10.2%)	0.003	0.35 (0.20,0.62)	0.25 (0.13,0.47)	0.23 (0.1,0.49)

*HR, the hazard ratio of in-hospital mortality between statin and non-statin (reference).

**Table 3:** Baseline characteristics of the study population by statin use in CHD or non-CHD population.

	Non CHD (N = 2885)				CHD (N = 248)			
	All	Statin (N = 279)	Non-Statin (N = 2606)	p	All	Statin (N = 125)	Non-Statin (N = 123)	p
Age	61 (20)	65 (15)	60 (20)	< 0.001	69.5 (13)	71 (13)	69 (15)	0.377
Sex	1463 (50.7%)	154 (55.2%)	1309 (50.2%)	0.115	109 (44%)	58 (46.4%)	51 (41.5%)	0.436
	1422 (49.3%)	125 (44.8%)	1297 (49.8%)		139 (56%)	67 (53.6%)	72 (58.5%)	
Hospital Stay	19.53 (18.8)	24.89 (25.14)	18.86 (18.1)	< 0.001	21.48 (19.76)	25.36 (24.87)	18.03 (15.66)	< 0.001
**Comorbidities**								
Hypertension	978 (33.9%)	138 (49.5%)	840 (32.2%)	< 0.001	181 (73%)	96 (76.8%)	85 (69.1%)	0.174
Dyslipidemia	22 (0.8%)	11 (3.9%)	11 (0.4%)	< 0.001	1 (0.4%)	1 (0.8%)	0 (0%)	0.322
Aortic Dissection	3 (0.1%)	1 (0.4%)	2 (0.1%)	0.165	1 (0.4%)	1 (0.8%)	0 (0%)	0.322
Heart Failure	21 (0.7%)	5 (1.8%)	16 (0.6%)	0.028	12 (4.8%)	8 (6.4%)	4 (3.3%)	0.25
Atrial fibrillation	11 (0.4%)	3 (1.1%)	8 (0.3%)	0.048	9 (3.6%)	6 (4.8%)	3 (2.4%)	0.322
Other arrhythmia	316 (11%)	38 (13.6%)	278 (10.7%)	0.134	17 (6.9%)	8 (6.4%)	9 (7.3%)	0.776
Diabetes milieus	342 (11.9%)	67 (24%)	275 (10.6%)	< 0.001	72 (29%)	38 (30.4%)	34 (27.6%)	0.634
Lung disease	174 (6%)	16 (5.7%)	158 (6.1%)	0.827	25 (10.1%)	11 (8.8%)	14 (11.4%)	0.501
Renal insufficiency	54 (1.9%)	8 (2.9%)	46 (1.8%)	0.197	15 (6%)	4 (3.2%)	11 (8.9%)	0.058
Stroke	90 (3.1%)	30 (10.8%)	60 (2.3%)	< 0.001	12 (4.8%)	8 (6.4%)	4 (3.3%)	0.25
Tumor	118 (4.1%)	7 (2.5%)	111 (4.3%)	0.161	11 (4.4%)	6 (4.8%)	5 (4.1%)	0.78
Liver disease	52 (1.8%)	2 (0.7%)	50 (1.9%)	0.152	3 (1.2%)	0 (0%)	3 (2.4%)	0.08
**Lab on admission**								
BNP (pg/ml)	81 (277.5)	91 (398)	80 (265.25)	0.826	326 (821.5)	445 (883)	224 (789)	0.855
Hs-cTnI (ng/ml)	3.8 (6.2)	5 (7.1)	3.6 (5.93)	0.675	8.1 (20.58)	8.1 (14.25)	8.2 (48.5)	0.181
ALT (u/l)	22 (24)	24 (21)	22 (24)	0.39	23 (24)	23 (23.5)	24 (25)	0.999
AST (u/l)	25 (18)	24 (16)	25 (18.25)	0.284	25 (24.75)	26 (17)	25 (27)	0.248
Creatinine (μmol/l)	68 (27)	68 (24)	68 (27)	0.631	74 (34)	73 (29)	74 (42)	0.875
K (mmol/L)	4.19 (0.64)	4.21 (0.75)	4.18 (0.64)	0.665	4.2 (0.78)	4.16 (0.83)	4.25 (0.76)	0.137
Na (mmol/L)	139.9 (4)	139.6 (4.8)	139.9 (4)	0.027	139.8 (5.07)	138.9 (6.25)	140.4 (4.6)	0.007
Ca (mmol/L)	2.16 (0.17)	2.16 (0.17)	2.16 (0.17)	0.529	2.14 (0.16)	2.13 (0.16)	2.15 (0.18)	0.742
Glucose (mmol/L)	5.87 (2.31)	6.15 (2.81)	5.85 (2.25)	0.004	6.28 (2.34)	6.22 (2.55)	6.37 (2.22)	0.924
ALB (g/L)	36.4 (8.4)	35.6 (7.5)	36.5 (8.4)	0.122	35 (7.38)	35.5 (7.5)	34.3 (7)	0.137
LDH (U/L)	247 (140.5)	247 (122)	246 (143)	0.267	263.5 (165.5)	265 (151.5)	251 (230)	0.045
Hb (g/l)	127 (22)	124 (21)	127 (22)	0.072	125.5 (24.75)	125 (21.5)	127 (27)	0.818
WBC (× 10^9^/l)	5.83 (3)	6.11 (2.92)	5.8 (2.98)	0.118	6.05 (3.03)	5.99 (2.79)	6.05 (3.02)	0.08
Lymph (× 10^9^/l)	1.21 (0.86)	1.23 (0.77)	1.21 (0.87)	0.836	1.04 (0.92)	1.09 (0.82)	0.98 (0.95)	0.569
PLT (× 10^9^/l)	222 (116)	232 (130)	220 (114.25)	0.005	214.5 (131)	214 (144.5)	215 (119)	0.327
hs_CRP (mg/l)	11.1 (57)	10 (51)	11.1 (57.33)	0.348	15.7 (55.4)	16.9 (53.6)	14.2 (55.7)	0.349
IL_2 (pg/ml)	519 (477.5)	501 (403)	520 (495.25)	0.053	609 (563.75)	570 (503)	653 (664)	0.059
IL_6 (pg/ml)	4.79 (19)	5.95 (14.88)	4.71 (19.59)	0.614	9.42 (22.95)	8.35 (21.15)	10.89 (28.09)	0.036
TNF-α (ng/ml)	7.9 (4.4)	8.1 (4.2)	7.9 (4.5)	0.255	8.95 (5.18)	8.8 (5.2)	9.1 (5.4)	0.341
PCT (ng/ml)	0.06 (0.07)	0.06 (0.05)	0.06 (0.07)	0.276	0.07 (0.11)	0.06 (0.1)	0.08 (0.13)	0.289
APTT (s)	38.7 (6.5)	38.5 (6)	38.7 (6.5)	0.102	38.45 (6.28)	38.5 (7.2)	38.4 (5.7)	0.769
PT_INR (s)	1.05 (0.12)	1.05 (0.14)	1.06 (0.12)	0.448	1.07 (0.12)	1.06 (0.1)	1.08 (0.17)	0.05
D_Dimer (μg/ml)	0.64 (1.38)	0.73 (1.4)	0.64 (1.35)	0.764	0.93 (1.76)	0.83 (1.43)	1.02 (2.14)	0.051
TC (mmol/L)	3.85 (1.24)	3.9 (1.48)	3.85 (1.2)	0.051	3.38 (1.27)	3.23 (1.04)	3.56 (1.52)	0.036
**7-days after admission**								
APTT (s)	38.2 (6)	38.1 (5.7)	38.2 (6.1)	0.215	38.55 (7.1)	39.1 (7.1)	38.4 (7.4)	0.656
PT_INR	1.04 (0.11)	1.04 (0.13)	1.04 (0.11)	0.193	1.06 (0.15)	1.05 (0.11)	1.06 (0.19)	0.372
D_Dimer (μg/ml)	0.49 (0.97)	0.51 (0.82)	0.49 (0.99)	0.028	0.79 (1.73)	0.61 (1.37)	0.96 (2.3)	0.004
hs_CRP (mg/l)	2 (7.4)	1.9 (5.3)	2.1 (7.53)	0.008	3.75 (28.23)	2.7 (13.6)	4.6 (53.9)	0.005
IL_2 (pg/ml)	443 (373)	429 (329)	446 (384.75)	0.011	540.5 (509.75)	508 (427.5)	594 (676)	0.072
IL_6 (pg/ml)	3.16 (7.01)	3.25 (6.52)	3.15 (7.06)	0.163	5.87 (21.19)	4.96 (11.08)	9.5 (40.74)	0.023
TNF-α (ng/ml)	7.7 (4.55)	8.1 (4.5)	7.6 (4.5)	0.63	8.65 (6.08)	8.8 (4.95)	8.4 (7.7)	0.16
TC (mmol/L)	4.26 (1.36)	4.11 (1.54)	4.28 (1.34)	0.082	3.56 (1.55)	3.4 (1.15)	3.98 (1.93)	0.064
**Lab Peak**								
IL2peak	545 (507)	518 (395)	547.5 (514.75)	0.011	657 (673.5)	624 (573.5)	679 (804)	0.118
IL6peak	5.99 (23.95)	6.85 (17.85)	5.94 (24.62)	0.15	11.52 (36.42)	10.52 (29.49)	11.69 (47.46)	0.02
TNFapeak	8.6 (4.8)	8.8 (4.3)	8.6 (5)	0.516	9.75 (5.95)	9.6 (4.95)	9.9 (6.7)	0.143
CRPpeak	13.5 (60.5)	13.5 (55.9)	13.5 (61.73)	0.218	24 (73.35)	27 (71.4)	19.4 (88.2)	0.175
**Medicine**								
Norepinephrine	202 (7%)	16 (5.7%)	186 (7.1%)	0.383	35 (14.1%)	12 (9.6%)	23 (18.7%)	0.04
Epinephrine	294 (10.2%)	19 (6.8%)	275 (10.6%)	0.05	48 (19.4%)	18 (14.4%)	30 (24.4%)	0.047
Dopamine	171 (5.9%)	12 (4.3%)	159 (6.1%)	0.226	29 (11.7%)	13 (10.4%)	16 (13%)	0.525
Dobutamine	33 (1.1%)	3 (1.1%)	30 (1.2%)	0.91	3 (1.2%)	2 (1.6%)	1 (0.8%)	0.573
Aspirin	171 (5.9%)	98 (35.1%)	73 (2.8%)	< 0.001	98 (39.5%)	74 (59.2%)	24 (19.5%)	< 0.001
Clopidogrel	73 (2.5%)	48 (17.2%)	25 (1%)	< 0.001	69 (27.8%)	53 (42.4%)	16 (13%)	< 0.001
Ticagrelor	5 (0.2%)	4 (1.4%)	1 (0%)	< 0.001	9 (3.6%)	8 (6.4%)	1 (0.8%)	0.019
Methylprednisolone	791 (27.4%)	68 (24.4%)	723 (27.7%)	0.23	82 (33.1%)	35 (28%)	47 (38.2%)	0.088
ACEI	69 (2.4%)	20 (7.2%)	49 (1.9%)	< 0.001	23 (9.3%)	15 (12%)	8 (6.5%)	0.137
ARB	281 (9.7%)	63 (22.6%)	218 (8.4%)	< 0.001	67 (27%)	47 (37.6%)	20 (16.3%)	< 0.001
Βeta-blocker	61 (2.1%)	12 (4.3%)	49 (1.9%)	0.008	30 (12.1%)	23 (18.4%)	7 (5.7%)	0.002
CCB	348 (12.1%)	51 (18.3%)	297 (11.4%)	0.001	67 (27%)	41 (32.8%)	26 (21.1%)	0.039
Non-invasive ventilator (%)	341 (11.8%)	39 (14%)	302 (11.6%)	0.24	34 (13.7%)	18 (14.4%)	16 (13%)	0.751
Invasive Ventilator (%)	256 (8.9%)	26 (9.3%)	230 (8.8%)	0.783	29 (11.7%)	12 (9.6%)	17 (13.8%)	0.303
ECMO (%)	26 (0.9%)	3 (1.1%)	23 (0.9%)	0.746	1 (0.4%)	1 (0.8%)	0 (0%)	0.322
**Type**								
1	860 (29.8%)	58 (20.8%)	802 (30.8%)	0.004	28 (11.3%)	11 (8.8%)	17 (13.8%)	0.761
2	1075 (37.3%)	118 (42.3%)	957 (36.7%)		100 (40.3%)	55 (44%)	45 (36.6%)	
3	950 (32.9%)	103 (36.9%)	847 (32.5%)		120 (48.4%)	59 (47.2%)	61 (49.6%)	

CHD, coronary heart disease; BNP, serum B-type natriuretic peptide; TNI, Troponin I; ALT, Alanine Aminotransferase; AST, Aspartate transaminase; Cr, Creatinine; ALB, Albumin; LDH, Lactate dehydrogenase; WBC, White blood cell; Lymph, Lymphocyte; Plt, Platelet; CRP, C Reactive Protein; IL-2, Interleukin 2; IL-6, Interleukin 6; TNF-α, P.C.T., Procalcitonin; Tumor necrosis factor-α; APTT, activated partial thromboplastin time; INR, international normalized ratio; TC, Total Cholesterol; ACEI, Angiotensin-converting-enzyme inhibitors; ARB, angiotensin receptor blockers; CCB, Calcium Channel Blocker; ECMO, Extracorporeal membrane oxygenation.

Continuous variables are presented as median (IQR), categorical variables are presented as n percentage.

**Table 4:** Baseline characteristics of the study population according to statin use in CHD or non-CHD population after PSM.

	Non-CHD (N = 502)				CHD (N = 102)			
	All	Statin (N = 275)	Non-Statin (N = 277)	p	All	Statin (N = 97)	Non-Statin (N = 95)	p
Age	65 (17)	64 (15)	65 (20)	0.701	68.5 (13.25)	71 (13)	67 (15.25)	0.29
**Sex**								
Male	266 (52.99%)	132 (55%)	134 (51.15%)	0.388	46 (45.1%)	32 (51.61%)	14 (35%)	0.102
Female	236 (47.01%)	108 (45%)	128 (48.85%)		56 (54.9%)	30 (48.39%)	26 (65%)	
Length of stay	21.69 (21.01)	24.02 (25.55)	20.33 (19.38)	0	22.02 (23.17)	26.51 (25.84)	18.35 (15.74)	0.016
**Comorbidities**								
Hypertension	217 (43.23%)	111 (46.25%)	106 (40.46%)	0.191	76 (74.51%)	47 (75.81%)	29 (72.5%)	0.712
Dyslipidemia	13 (2.59%)	5 (2.08%)	8 (3.05%)	0.495	0 (0%)	0 (0%)	0 (0%)	——
Aortic Dissection	3 (0.6%)	1 (0.42%)	2 (0.76%)	0.615	1 (0.98%)	1 (1.61%)	0 (0%)	0.425
Heart Failure	9 (1.79%)	4 (1.67%)	5 (1.91%)	0.839	3 (2.94%)	2 (3.23%)	1 (2.5%)	0.834
Atrial fibrillation	6 (1.2%)	3 (1.25%)	3 (1.15%)	0.914	5 (4.9%)	2 (3.23%)	3 (7.5%)	0.334
Other arrhythmia	51 (10.16%)	34 (14.17%)	17 (6.49%)	0.004	5 (4.9%)	3 (4.84%)	2 (5%)	0.971
Diabetes milieus	100 (19.92%)	47 (19.58%)	53 (20.23%)	0.857	31 (30.39%)	18 (29.03%)	13 (32.5%)	0.713
Lung disease	31 (6.18%)	11 (4.58%)	20 (7.63%)	0.157	10 (9.8%)	6 (9.68%)	4 (10%)	0.958
Renal insufficiency	15 (2.99%)	8 (3.33%)	7 (2.67%)	0.664	5 (4.9%)	2 (3.23%)	3 (7.5%)	0.334
Stroke	37 (7.37%)	19 (7.92%)	18 (6.87%)	0.655	4 (3.92%)	1 (1.61%)	3 (7.5%)	0.137
Tumor	17 (3.39%)	7 (2.92%)	10 (3.82%)	0.578	6 (5.88%)	3 (4.84%)	3 (7.5%)	0.581
Liver disease	4 (0.8%)	2 (0.83%)	2 (0.76%)	0.93	1 (0.98%)	0 (0%)	1 (2.5%)	0.215
**Lab on admission**								
BNP (pg/ml)	97 (345)	85.5 (339)	118.5 (342.75)	0.735	248.5 (759.25)	315.5 (697)	223.5 (795.5)	0.876
Hs-cTnI (ng/ml)	4.8 (7.13)	4.7 (6.68)	5 (7.43)	0.145	7.95 (14.45)	7.55 (7.7)	8.15 (27.95)	0.133
ALT (u/l)	24 (24)	24.5 (21)	22 (26.5)	0.582	25.5 (26.25)	23.5 (25.5)	26 (29.25)	0.816
AST (u/l)	25 (19)	24 (16)	25 (20)	0.174	25.5 (20)	25.5 (16.75)	25.5 (19)	0.814
Creatinine (μmol/l)	68 (25.25)	68.5 (25.75)	68 (26)	0.486	70 (34.5)	69 (25.5)	73.5 (49)	0.22
K (mmol/L)	4.19 (0.71)	4.23 (0.79)	4.16 (0.67)	0.633	4.09 (0.82)	4.19 (0.9)	3.97 (0.65)	0.744
Na (mmol/L)	139.6 (4.43)	139.8 (4.75)	139.5 (4.2)	0.879	139.4 (5.68)	139 (6.55)	140.35 (4.32)	0.428
Ca (mmol/L)	2.16 (0.17)	2.17 (0.17)	2.16 (0.18)	0.533	2.13 (0.16)	2.13 (0.16)	2.13 (0.16)	0.339
Glucose (mmol/L)	6.08 (2.51)	6.06 (2.55)	6.09 (2.37)	0.822	6.13 (2.21)	6.05 (2.54)	6.19 (2.01)	0.225
ALB (g/L)	35.65 (7.83)	35.9 (7.78)	35.3 (7.93)	0.382	35.1 (7.05)	35.6 (6.63)	33.65 (8.3)	0.311
LDH (U/L)	256 (130)	241.5 (104.75)	265 (158.5)	0.071	248.5 (168)	263 (168.25)	225 (174.75)	0.609
Hb (g/l)	126 (22)	125 (20.75)	126 (24)	0.79	124 (24.25)	123 (23.25)	128 (25.75)	0.548
WBC (× 10^9^/l)	6.04 (2.91)	6.07 (2.88)	6.03 (2.81)	0.407	5.97 (2.77)	6.02 (2.96)	5.69 (2.59)	0.961
Lymph (× 10^9^/l)	1.21 (0.82)	1.29 (0.86)	1.14 (0.85)	0.034	1.11 (0.9)	1.17 (0.87)	1.04 (0.93)	0.979
PLT (× 10^9^/l)	229.5 (128.5)	231 (126.5)	228.5 (127.25)	0.498	216.5 (136.75)	219 (145)	205 (107.75)	0.582
hs_CRP (mg/l)	13.6 (58.83)	8.95 (51.05)	20.15 (67.8)	0.245	16.15 (44.95)	17 (66.2)	13.95 (36.48)	0.548
IL_2 (pg/ml)	534.5 (493.25)	493 (408.75)	566 (584.5)	0	625.5 (470.75)	612 (424.75)	677.5 (530.25)	0.278
IL_6 (pg/ml)	6 (18.5)	5.55 (13.99)	6.55 (23.87)	0.586	9.08 (23.3)	8.18 (23.29)	10.12 (25.41)	0.236
TNF-α (ng/ml)	8.2 (4.3)	8 (4.18)	8.5 (4.33)	0.042	9.25 (4.9)	8.9 (5.2)	9.85 (4.58)	0.789
PCT (ng/ml)	0.07 (0.07)	0.06 (0.05)	0.07 (0.07)	0.886	0.07 (0.09)	0.06 (0.1)	0.08 (0.09)	0.956
APTT (s)	38.7 (6.8)	38.5 (6.1)	38.75 (7.33)	0.294	38.5 (6.8)	38.75 (6.68)	38.1 (6.08)	0.099
PT_INR (s)	1.06 (0.13)	1.05 (0.13)	1.06 (0.12)	0.222	1.07 (0.13)	1.07 (0.1)	1.07 (0.18)	0.991
D_Dimer (μg/ml)	0.77 (1.6)	0.64 (1.41)	0.89 (1.71)	0.775	0.81 (1.39)	0.81 (1.34)	0.8 (1.69)	0.336
TC (mmol/L)	3.85 (1.31)	3.91 (1.45)	3.81 (1.17)	0.045	3.21 (1.26)	3.13 (1.21)	3.39 (1.67)	0.527
**7-days after admission**								
APTT (s)	38.3 (6.3)	38.1 (5.98)	38.4 (6.9)	0.073	38.8 (6.9)	38.95 (6.45)	38.4 (9.4)	0.959
PT_INR	1.04 (0.12)	1.04 (0.13)	1.04 (0.11)	0.175	1.04 (0.11)	1.04 (0.09)	1.04 (0.25)	0.428
D_Dimer (μg/ml)	0.52 (0.99)	0.48 (0.74)	0.59 (1.32)	0.012	0.71 (1.4)	0.61 (1.26)	0.81 (1.93)	0.166
hs_CRP (mg/l)	2.1 (5.6)	1.9 (5.03)	2.3 (7.33)	0.012	2.5 (15.48)	2.35 (8.8)	2.55 (35.33)	0.646
IL_2 (pg/ml)	452 (356.5)	409 (326.75)	493.5 (431.75)	0	502.5 (494.25)	470 (416.75)	587 (610)	0.103
IL_6 (pg/ml)	3.43 (6.58)	2.96 (6.29)	4.01 (7.92)	0.653	4.48 (11.91)	3.75 (8.72)	7.48 (25.68)	0.061
TNF-α (ng/ml)	8.05 (4.2)	8 (4.28)	8.1 (4.2)	0.774	9.45 (5.65)	9.15 (5.4)	9.45 (7.05)	0.246
TC (mmol/L)	4.12 (1.54)	4.11 (1.61)	4.14 (1.53)	0.741	3.52 (1.32)	3.46 (1.23)	3.88 (1.44)	0.586
**Lab Peak**								
IL2peak	551.5 (510.75)	514 (414)	596.5 (629.75)	0	654.5 (591.75)	637.5 (491.75)	702.5 (769)	0.317
IL6peak	6.88 (20.89)	6.54 (15.41)	8.1 (28)	0.433	10.29 (36.6)	10.29 (35.54)	10.28 (37.71)	0.07
TNFapeak	8.8 (4.43)	8.7 (4)	9 (4.9)	0.394	10.05 (5.45)	9.9 (5.05)	10.25 (5.9)	0.448
CRPpeak	17.25 (62)	12.95 (53.5)	23.95 (69.13)	0.137	20.25 (65.2)	27.3 (77.55)	15.9 (38.8)	0.343
**Medicine**								
Norepinephrine	32 (6.37%)	11 (4.58%)	21 (8.02%)	0.116	6 (5.88%)	1 (1.61%)	5 (12.5%)	0.022
Epinephrine	48 (9.56%)	13 (5.42%)	35 (13.36%)	0.002	13 (12.75%)	5 (8.06%)	8 (20%)	0.079
Dopamine	27 (5.38%)	8 (3.33%)	19 (7.25%)	0.052	7 (6.86%)	4 (6.45%)	3 (7.5%)	0.84
Dobutamine	9 (1.79%)	3 (1.25%)	6 (2.29%)	0.381	1 (0.98%)	1 (1.61%)	0 (0%)	0.425
Aspirin	137 (27.29%)	66 (27.5%)	71 (27.1%)	0.92	46 (45.1%)	23 (37.1%)	23 (57.5%)	0.044
Clopidogrel	50 (9.96%)	26 (10.83%)	24 (9.16%)	0.533	37 (36.27%)	21 (33.87%)	16 (40%)	0.534
Ticagrelor	3 (0.6%)	2 (0.83%)	1 (0.38%)	0.513	3 (2.94%)	2 (3.23%)	1 (2.5%)	0.834
Methylprednisolone	126 (25.1%)	58 (24.17%)	68 (25.95%)	0.645	32 (31.37%)	19 (30.65%)	13 (32.5%)	0.846
ACEI	22 (4.38%)	13 (5.42%)	9 (3.44%)	0.28	11 (10.78%)	6 (9.68%)	5 (12.5%)	0.657
ARB	87 (17.33%)	46 (19.17%)	41 (15.65%)	0.299	30 (29.41%)	20 (32.26%)	10 (25%)	0.437
Βeta-blocker	13 (2.59%)	6 (2.5%)	7 (2.67%)	0.904	10 (9.8%)	9 (14.52%)	1 (2.5%)	0.047
CCB	83 (16.53%)	40 (16.67%)	43 (16.41%)	0.939	29 (28.43%)	21 (33.87%)	8 (20%)	0.132
Non-invasive ventilator (%)	65 (12.95%)	33 (13.75%)	32 (12.21%)	0.609	16 (15.69%)	7 (11.29%)	9 (22.5%)	0.131
Invasive Ventilator (%)	38 (7.57%)	21 (8.75%)	17 (6.49%)	0.34	12 (11.76%)	6 (9.68%)	6 (15%)	0.42
ECMO (%)	4 (0.8%)	2 (0.83%)	2 (0.76%)	0.93	1 (0.98%)	1 (1.61%)	0 (0%)	0.425
**Type**								
1	134 (26.69%)	53 (22.08%)	81 (30.92%)	0.421	11 (10.78%)	5 (8.06%)	6 (15%)	0.827
2	182 (36.25%)	102 (42.5%)	80 (30.53%)		45 (44.12%)	30 (48.39%)	15 (37.5%)	
3	186 (37.05%)	85 (35.42%)	101 (38.55%)		46 (45.1%)	27 (43.55%)	19 (47.5%)	

CHD, coronary heart disease; BNP, serum B-type natriuretic peptide; TNI, Troponin I; ALT, Alanine Aminotransferase; AST, Aspartate transaminase; Cr, Creatinine; ALB, Albumin; LDH, Lactate dehydrogenase; WBC, White blood cell; Lymph, Lymphocyte; Plt, Platelet; CRP, C Reactive Protein; IL-2, Interleukin 2; IL-6, Interleukin 6; TNF-α, P.C.T., Procalcitonin; Tumor necrosis factor-α; APTT, activated partial thromboplastin time; INR, international normalized ratio; TC, Total Cholesterol; ACEI, Angiotensin-converting-enzyme inhibitors; ARB, angiotensin receptor blockers; CCB, Calcium Channel Blocker; ECMO, Extracorporeal membrane oxygenation.

Continuous variables are presented as median (IQR), categorical variables are presented as n percentage.

## Discussion

In this retrospective study, statin use was associated with a lower risk of all-cause in-hospital mortality than the non-statin group among COVID-19 patients. The benefit was significant both among CHD patients and non-CHD patients. Although unmeasured confounding and indication bias may have contributed to the observed protective association, these data showed that statin use was associated with survival benefits in COVID-19. These findings provide additional clinical evidence supporting continuous statin use in CHD patients with COVID-19, both in CHD and non-CHD patients. Given this study's retrospective nature, these data need further validation in randomized controlled trials to determine the efficacy of statin use in patients and COVID-19.

Due to the conflicting considerations of statin use during COVID-19, several studies examined statin use during COVID-19. A study in China showed a survival benefit in statin users vs. non-statin users^17^. A most recent study examined diabetes patients with COVID-19 and showed that statin use was associated with reduced in-hospital mortality from COVID-19 in diabetes mellitus patients^3^. Such benefits did not show in non-diabetic patients. In diabetes or hyperglycemic status, virus entrance and replication are intensified. COVID-19 leads to inflammatory/oxidative stress and exaggerate cytokine storm, which is particularly enhanced in diabetes^12–14^. In COVID-19, serum concentrations of proinflammatory cytokines, including IL-2, IL-6, and tumor necrosis factor (TNF)-a, were markedly increased in severe cases than moderate cases, suggesting that cytokine storms could be associated with disease severity. Statin was known for its anti-inflammatory quality. Diabetes and cardiovascular disease were two major comorbidities that increased the mortality of COVID-19^2,18,19^, yet there is no study examining statin use in CHD patients. Also, our study examined the change of inflammatory marker levels at presentation, seven days post-admission, and their peak levels both in statin and non-statin groups among CHD patients.

CHD is one of the most prevalent comorbidities in COVID-19 patients. Our COVID-19 population has a CHD prevalence of 7.9%, which echoes the previous COVID-19 report in Wuhan, and was lower than US studies^6^. The definition of CHD in our study was those who had a medical record shown CHD as one of the discharge diagnoses. In such case, CHD included all patients with coronary heart disease, such as those who had a history of CHD, treated with PCI, CAGB, or receiving medical therapy, as long as clinicians have once diagnosed it. The individual case fatality rate of COVID-19 patients with CHD was 10.5%, highest among those with any comorbidities, including chronic respiratory disease (6.3%), cancer (5.6%), diabetes (7.3%), and hypertension (6.0%)^20^. CHD predispose patients with proinflammatory and procoagulant status, together with COVID-19, increased the risk of thrombus formation, severe inflammatory response, and endothelial damage, leading to ACS, heart failure, cardiac arrhythmia, and sudden death^21–23^. COVID-19 can also directly trigger ACS by plaque rupture, coronary spasm, or microthrombi due to systemic inflammation or cytokine storm^24^. Meanwhile, the most common comorbidities associated with CHD, such as hypertension increased ACE2 receptor and facilitated virus entry, diabetes enhanced cytokine storm, and leads to multi-organ failure^10,11,25^. Moreover, COVID-19 was associated with hypercoagulation status and could cause increased intracoronary thrombosis and worsen STEMI outcomes^26^. Therefore, COVID-19 patients with CHD were four times more likely to die than average COVID-19 patients, with an elevated level of proinflammatory cytokines such as IL-6, CRP, and procoagulant factors such as D-Dimer^20^.

Statin is known for its anti-inflammatory and anticoagulant effects^27^. SARS-CoV-2 is targeting the endothelium, one of the largest organs in the human body. Notably, endothelial cells express all the co-factors necessary for the internalization of SARS-CoV-2 in human host cells^28,29^. Moreover, the most common comorbidities in COVID-19 such as hypertension and diabetes were generally predisposed with endothelial dysfunction, which promotes virus entry, replication, clotting cascade, microvascular obstruction and multi-organ failure^25^. Dysregulated RAS system in CHD patients also increases ACE2 expression, results in pre-existing endothelial dysfunction which facilitates virus entry, finally causees subsequent cardiac damages^29–31^. On the other hand, SARS-CoV-2 per se induces cytokine storm, which further exacerbates endothelial injury and dysfunction, ultimately leading to microvascular thrombi, pro-thrombotic state, and cardiovascular ischemia status^25,29^. Expert in bioengineering and nanomedicine have tried to develop numerous innovative pharmaceutical approaches targeting endothelial dysfunction to tackle this problem^28^. Statin is one of the most promising drug classes for treating endothelial dysfunction and preventing vascular damage in COVID-19. Similar to RAS inhibitors, statins improve endothelial function in patients with or at risk for cardiovascular disease. They improve endothelial function via reduction of LDL and suppression of pro-oxidant enzymes and other proinflammatory transcriptional and signal transduction pathways^32^. Previous studies showed that statin protected diabetic COVID-19 patients from in-hospital mortality by anti-inflammatory properties and antiplatelet effect^3^. Its acute administration improves survival in proinflammatory states such as myocardial infarction. Our study shows that statin use in CHD patients vastly improved in-hospital morality, with reduced IL-2, IL-6 level, and D-Dimer levels during the hospital stay.Similarly, such benefits are also seen in non-CHD patients and more critically ill patients. To avoid an unbalanced baseline, we used propensity score match to retest our result and yield a similar conclusion of statin benefit. However, due to this study's retrospective nature, a prospective design is warranted to prove the conclusion further.

## Limitation

Our study should be viewed considering several limitations inherent in retrospective analyses. First, although we had detailed information on medical history, our observational study cannot exclude the potential impact of unmeasured confounders such as social status, the duration of statin use, and the reason for statin use. Second, we did not examine pre-hospital statin use. Such information was missed in the electronic data collection system because of the urgent COVID-19 time. However, we defined statin use as patients received statin within 3-day post-hospitalization. We choose this definition to include the statin group was patients who were regular statin users. Also, it has not been our routine practice for clinicians to initiate new statin therapy in patients with acute COVID-19 illness. In addition, clinical scenarios leading to statin cessation such as liver enzyme elevation were not significantly different between the statin and non-statin groups. Meanwhile, our data showed only two patients who initiated statin after three-day post-hospitalization. Both of them were fully recovered and discharged. As a result, excluding the two late-use statin cases may have underestimated the benefit of statin treatment on COVID-19 patients but would not change the direction of our results. Third, due to all patients were hospitalized with COVID-19 in Wuhan, generalizability may be limited. Finally, due to the observational study's inherent limitation, our study did not examine the causality between statin use and reduced in-hospital mortality, which should be tested only by a randomized control trial.

## Conclusion

Among patients hospitalized with COVID-19, statin use was associated with reduced in-hospital mortality from COVID-19. The benefit of statin was both prominent among patients with CHD and non-CHD patients. These findings may further reemphasize the continuation of statins to patients with CHD during the COVID-19 era.

### Ethical approval

All procedures performed in studies involving human participants were following the ethical standards of the institutional and/or national research committee and with the 1964 Helsinki declaration and its later amendments or comparable ethical standards.

### Informed consent

This study was approved by the Ethics Commission of Tongji Hospital. Written informed consent was waived for the retrospective study by the Ethical Committee of Tongji Hospital, Tongji Medical College, Huazhong University of Science and Technology.

### Consent for publication

All authors have also reviewed and approved the manuscript.

## Data Availability

The datasets generated during and/or analyzed during the current study are available from the corresponding author on reasonable request.
